# How Healthy Are Our Students Really? Lifestyle-Related Health Risk Behaviour Patterns in Student Athletes and Nonathlete Students

**DOI:** 10.1155/tsm2/1994649

**Published:** 2025-03-20

**Authors:** Gabriella Florence, Melissa Janse van Vuren, Wayne Derman, Jake Popperwell, Liske Kotzé-Hörstmann

**Affiliations:** ^1^Institute of Sport and Exercise Medicine, Department of Exercise, Sport and Lifestyle Medicine, Stellenbosch University, Cape Town, South Africa; ^2^International Olympic Committee Research Centre, Johannesburg, South Africa

**Keywords:** alcohol, cigarette smoking, cluster analysis university student athletes, dietary habits, physical activity

## Abstract

**Introduction:** Students engage in one or more lifestyle-related risk behaviours that may predispose them to noncommunicable diseases (NCDs). Whilst student athletes (St-A) are often perceived as having healthier lifestyles compared to nonathlete students (NAS), it is unclear whether they exhibit better risk profiles. This study compares the prevalence and clustering of these lifestyle-related risk behaviours among NAS and St-A at a South African university.

**Methods:** This cross-sectional study assessed the prevalence of alcohol consumption, cigarette smoking, inadequate fruit and vegetable intake and physical inactivity using a screening questionnaire. Differences between gender and athletic status were analysed using chi-square tests. Cluster analysis was employed to identify patterns of co-occurring risk behaviours within the combined cohort.

**Results:** One hundred and forty-five St-A (*n* = 91 male, *n* = 54 female) and 143 NAS (*n* = 90 male, *n* = 53 female) were included. Similar alcohol use patterns were observed between St-A and NAS (*p*=0.737), but females consumed less alcohol than men (*p*=0.025). Smoking was less prevalent among St-A (18.6% vs. 32.2% NAS, *p*=0.020). Less than 10% of participants met fruit and vegetable intake recommendations, with females consuming more than males (*p*=0.002). St-A met the moderate activity guidelines more often (73.0% vs. 44.3%, *p* < 0.001) and the vigorous physical activity guidelines more often (71.5% vs. 37.1%, *p* < 0.001) than NAS. The cluster analysis identified a higher risk group (34.6% St-A) with multiple risk behaviours, and a healthier group (all meeting vigorous activity goals and none smoking).

**Conclusions:** Both St-A and NAS engage in lifestyle-related risk behaviours. Further investigation into the interconnections of these behaviours and the implementation of university-based interventions is warranted.

## 1. Introduction

The transition from school to university is a critical period for young adults, marked by significant social, physical and emotional changes [1]. During this time, students may adopt lifestyle-related risk behaviours such as excessive alcohol consumption, cigarette smoking, poor dietary choices and reduced physical activity levels [2]. These behaviours, if persistent, can lead to noncommunicable diseases (NCDs) such as heart disease, diabetes and certain cancers, and premature mortality [3]. In South Africa, studies reveal prevalent lifestyle-related risk behaviours among youth, including excessive alcohol consumption, physical inactivity and smoking [4]. These behaviours often continue postgraduation into late adulthood [5, 6].

Some studies suggest student athletes (St-A; concurrently completing an academic degree and competing in a sport at a university level) are less likely to engage in lifestyle-related risk behaviours and are less susceptible to metabolic risk factors such as being overweight [7, 8] compared to nonathlete students (NAS). However, Divin [9], Nattiv et and Wechsler et al. [10–12] suggested that due to the increased pressure to succeed in both sport and academics, St-A may be more prone to engaging in lifestyle-related risk behaviours such as heavy drinking, smoking and inadequate dietary practices [13]. This is surprising because engagement in excessive alcohol consumption has shown to impair sports performance by about 11.4% [14], cigarette smoking hinders athletic performance by adversely affecting cardiopulmonary functions and maximal oxygen uptake [15, 16] and suboptimal nutrition can lead to metabolic dysfunction including low energy availability (LEA), reduced bone density and weakened immune function [17].

Multiple lifestyle-related risk behaviours often occur together and synergistically increase the likelihood of developing NCDs beyond the sum of their individual effects [18, 19]. Moreover, these combined risk factors can negatively affect mental health and further increase the risk of premature mortality and diminish quality of life [20]. This highlights the importance of designing holistic health interventions that address multiple lifestyle-related risk behaviours to reduce the risk of NCDs rather than targeting them in isolation. This approach is not only vital for reducing the risk and burden of NCDs later in life but can also improve academic and sports performance.

Previous studies on the prevalence of health and lifestyle-related risk behaviours in South African universities have been limited to the Gauteng province and have thus far focused on either St-A or NAS [21, 22]. Moreover, co-occurrence of these lifestyle-related risk behaviours in the student and athlete populations has never been investigated. Therefore, this study aimed to compare lifestyle-related risk behaviours, including excessive alcohol consumption, smoking, low fruit and vegetable intake and physical inactivity, among St-A and NAS residing in the Western Cape, South Africa. In addition, considering the diverse and interconnected nature of these behaviours, this study includes a cluster analysis to identify distinct subgroups based on their lifestyle-related risk behaviours. This analysis may reveal patterns of lifestyle-related risk behaviour clustering, providing insights for targeted university-level intervention strategies.

## 2. Materials and Methods

### 2.1. Study Design

This cross-sectional descriptive study analysed health- and lifestyle-related risk behaviour data collected from the *Maties Risk of Nonommunicable Diseases* (MaRooN) Health Passport between March and November 2021 from St-A and NAS (collectively referred to as “participants”) registered at a university in the Western Cape, South Africa. The MaRooN Health Passport is a campus-wide health surveillance system developed by the Institute of Sport and Exercise Medicine (ISEM) and Campus Health Services at Stellenbosch University that aims to monitor and manage the health and well-being profiles of the university staff and student population.

### 2.2. Study Setting and Population

The St-A included in this study were recipients of competitive university bursaries based on athletics (sporting) ability, who participated in university-level competitive sports. No exclusion criteria were applied. One hundred and forty-five St-A (*n* = 91 male and *n* = 54 female) older than 18 years completed the MaRooN Health Passport. A group of 143 NAS (*n* = 90 male and *n* = 53 female) older than 18 years who also completed the MaRooN Health Passport were selected from the MaRooN Health Passport database as the comparator group. Using Microsoft Excel to generate a random selection of students, this NAS group was matched for age, faculty and self-reported gender to the St-A group. This ensured equal numbers of age-matched male and female participants as well as comparative representations from the different university faculties.

### 2.3. Data Collection

The participant demographic information extracted from the MaRooN Health Passport database included self-reported gender, faculty, age, and year of study. The health and lifestyle-related risk behaviours were frequency of alcohol consumption (“*How often do you have a drink containing alcohol? Never, Monthly or less, 2-4 times a month, 2-3 times a week, Daily or almost daily”*), frequency of smoking cigarettes (“*How often do you smoke? Never, Monthly or less, 2-4 times a month, 2-3 times a week, Daily or almost daily*”) and number of fruit and vegetable servings consumed per day (*“Fruit and vegetables intake. None, 1-2, 3-4, ≥ 5 servings”*) [23]. Responses to these questions were coded to Likert-type response variables (0, 1, 2, 3 and/or 4) for subsequent data analysis. Physical activity levels were recorded as number of days per week, and hours per day spent doing vigorous (e.g., heavy lifting, digging, aerobics or fast bicycling) and moderate physical activity (e.g., carrying light loads, bicycling at a regular pace or doubles tennis) [24]; these data were used to calculate inadequate moderate physical activity (< 600 metabolic equivalent of task [MET] minutes per week) or inadequate vigorous physical activity (< 1500 MET minutes per week) [25].

### 2.4. Data Analysis

Descriptive statistics were used to describe the participants' characteristics for age, self-reported gender and year of study. The lifestyle-related health-risk behaviours were individually analysed and presented as prevalence percentages for alcohol consumption (frequency), cigarette smoking (frequency), consuming less than five daily servings of fruits and vegetables and physical inactivity (inadequate moderate and vigorous physical activities). The data were stratified by gender and athlete status. Group differences in the lifestyle-related risk behaviours between NAS and St-A groups, stratified by gender, were evaluated using Pearson's chi-square (*χ*^2^), and Bonferroni adjustment for multiple tests.

To investigate the co-occurrence and patterns of lifestyle-related risk behaviours among St-A and NAS, a two-step cluster analysis was conducted. Alcohol use frequency, smoking habits and fruit and vegetable consumption were quantified using Likert scale coding, whilst levels of moderate and vigorous physical activities were converted into binary variables (*meeting WHO guidelines* = 0, *not meeting WHO guidelines* = 1) for categorical analysis. The lifestyle-related risk behaviours (alcohol consumption, cigarette smoking, inadequate fruit and vegetable intake and inadequate moderate and inadequate vigorous physical activity) were included in the model, whilst demographic variables such as age and gender were included as evaluation fields but not as clustering variables. The optimal number of clusters was determined autonomously utilising the Bayesian information criterion for data-driven determination of the cluster structure. The robustness and appropriateness of the clustering solution were validated using silhouette measures. This method quantitatively assessed the similarity of each case within its assigned cluster relative to other clusters, ensuring a reliable and statistically sound validation of the cluster analysis. Significant differences in lifestyle-related risk behaviours and demographic descriptors between clusters were calculated with Pearson's chi-square (*χ*^2^). The *p* value (two-sided) less than 0.05 was considered significant. All statistical analyses were performed using IBM Statistical Package for the Social Sciences (SPSS) (IBM Corporation, Armok, USA; Version 27).

### 2.5. Ethical Considerations

Ethical approval for this study was obtained from the Research Ethics Committee: Social, Behavioural and Education Research at Stellenbosch University (REC-SBE: 25,297). The protocol conformed to the recommendations of the Declaration of Helsinki and was also approved by the Institutional Research Office. Electronic informed consent for data to be used in the research was obtained from all participants.

## 3. Results

### 3.1. Participants

As part of the university's sports onboarding process, 184 sports bursary recipients were sent a link with a QR code and invitation to complete the MaRooN survey. Of the 184 St-A who opened the link for the MaRooN Health Passport, 145 completed it (*n* = 91 male and *n* = 54 female; mean age 22.1 ± 2.1 years), equating to a 78.8% response rate. From a total of 1049 NAS who completed the MaRooN Health Passport (60.3% response rate), 143 NAS were selected to match for gender and faculty (*n* = 90 male and *n* = 53 females; mean age 23.5 ± 4.4 years). Accordingly, the age distribution between St-A and NAS was similar, with the majority of individuals in both groups being between the ages of 20 and 24 years. Most St-A (*n* = 122, 84%) reported involvement in team sports at the university competitive level, 65 (45%) participating in individual sports and 82 (56%) involved in other sporting activities. In contrast, NAS primarily participated in other activities (*n* = 134, 94%), followed by individual sports (*n* = 62, 43%) and team sports (*n* = 42, 29%) at a recreational level (Supporting Table 1).

### 3.2. Prevalence of Lifestyle-Related Risk Behaviours

The prevalence of lifestyle-related risk behaviours among St-A and NAS is presented in Table 1. Males consumed alcohol more frequently than females (*p*=0.025), although no significant differences were noted in alcohol consumption frequencies between St-A and NAS (*p* > 0.737). Around a quarter of participants reported regular tobacco use, with comparable prevalence between genders (28.7% of males vs. 19.6% of females, *p*=0.523). St-A, however, had a lower smoking prevalence (18.6%) compared to NAS (32.2%, *p*=0.020). Notably, male NAS had a lower proportion of nonsmokers and higher rates of daily smoking compared to their athlete counterparts (*p*=0.022). The difference was not observed among female students.

Fruit and vegetable consumption was generally low in both groups, with only 9.0% of St-A and 7.7% of NAS consuming five or more daily servings. The majority of students in both groups (approximately two-thirds), consumed one to two servings or less daily. Female students consume significantly more fruits and vegetables daily compared with males (*p*=0.002).

Levels of physical activity were markedly different between St-A and NAS. For moderate physical activity, 50.6% of male and 64.2% of female NAS did not meet physical activity guidelines, significantly higher than 24.7% of male St-A and 30.8% of female St-A. In terms of vigorous physical activity, 56.3% of male and 73.6% of female NAS fell short of the physical activity guidelines, compared to only 24.7% of male and 34.6% of female St-A, highlighting a notable but expected gap in physical activity levels between St-A and NAS.

### 3.3. Clustering of Lifestyle-Related Risk Behaviours Among St-A and NAS

**Figure 1:**
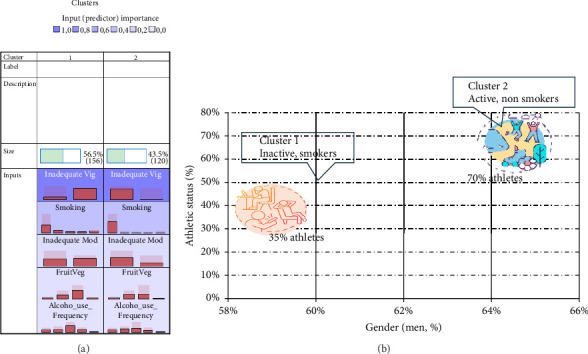
Clustering of lifestyle-related risk behaviours in a sample of university level St-A and NAS students. (a) A cluster model showing the input variables (predictors) ranked by influence on group membership assignment from most- to least-important. (b) Graphic representation of the proportions of the study participants by gender (% men) and athletic status (% student athletes) in Cluster 1 and Cluster 2. The reference categories for gender represent the percentage of men, whilst for athletic status, they represent the percentage of St-A in each cluster. NAS, nonathlete students; St-A, student athletes.

To evaluate the co-occurrence of lifestyle-related risk factors in St-A and their nonathlete counterparts, a two-way cluster analysis was employed (Figure 1). The purpose of this analysis was to identify natural groupings based on shared lifestyle-related risk behaviours among all participants in this cohort. Two significantly different clusters were identified. Not meeting guidelines for vigorous physical activity was the most significant predictor of cluster membership, followed by smoking status, inadequate moderate physical activity and fruit and vegetable consumption. Alcohol use frequency had a smaller impact on the cluster solution and group membership. The prevalence/distribution of lifestyle-related risk behaviours and demographic descriptors between the two clusters is shown in Table 2. Cluster 1 was characterised as the higher risk group based on the co-existence of several lifestyle factors. A significant portion of this cluster did not meet WHO vigorous activity goals (80.8%), with nearly half also falling short of moderate physical activity recommendations (51.3%). The higher risk group had a substantial number of individuals who consumed alcohol more regularly, with only 14.1% being non drinkers and 43.6% frequently consuming alcohol. In terms of smoking, 54.5% were classified as nonsmokers, whilst 29.8% engaged in either regular or heavy smoking. For fruit and vegetable intake, the majority (59.6%) had a low (1–2 servings) consumption, although 6.4% did not report consuming any fruit and vegetables regularly. This cluster contains a mix of St-A (34.6%) and NAS (65.4%). Cluster 2 was designated as the healthier group, with all members meeting vigorous physical activity goals and the majority also meeting moderate physical activity goals (71.7%). This cluster was characterised by less substance use, with 100% of participants designated as nonsmokers and 20% denied any alcohol consumption. In terms of diet, a greater proportion of this cluster consumed low (45.8%) to moderate (41.7%) amounts of fruits and vegetables compared to Cluster 1. Notably, Cluster 2 consisted of a higher proportion of St-A (69.2%) compared to NAS (30.8%). Both clusters had no significant differences in gender distribution or age groups, suggesting that these demographic factors do not distinctly differentiate these clusters in terms of the lifestyle-related risk factors analysed.

## 4. Discussion

This study investigated the prevalence of lifestyle-related risk behaviours in South African St-A compared to a comparator group of NAS, specifically alcohol consumption, cigarette smoking, inadequate fruit and vegetable consumption and physical inactivity. This study shows that whilst St-A engage in higher levels of physical activity and have lower rates of smoking, they exhibit similar patterns of alcohol consumption and inadequate fruit and vegetable intake, compared to their NAS counterparts. Females, regardless of their athletic status, were less likely to smoke and consume alcohol compared to males.

Furthermore, our findings reveal a significant co-occurrence of physical inactivity, smoking and suboptimal nutrition among all students which reflects the interconnected nature of these lifestyle-related risk behaviours. It is commonly perceived that athletic status offers a protective effect against engaging in lifestyle-related risk behaviours among university students [8, 26, 27]. Consequently, St-A may not be considered to be at comparable risk of these behaviours as their nonathlete peers. This mistaken assumption could lead to their omission by healthcare practitioners and policymakers in the university setting, with respect to the development of screening initiatives and intervention strategies.

### 4.1. Physical Activity

St-A are considered to maintain high levels of physical activity which deems them “healthier” than their nonathlete counterparts [26–28]. Indeed, our study shows that the percentage of NAS not meeting recommendations for vigorous and moderate physical activity were more than twice that of St-A. Notably, however, one-quarter of St-A also reported inadequate vigorous and moderate physical activities. This may be owing to certain sports not attaining the threshold for vigorous or moderate physical activity (such as golf, cricket or archery) [29]. Our findings support other international studies which also observed that St-A participate in more physical activity than NAS [26, 30]. Miller et al. observed that St-A were four times and twice as likely to engage in vigorous and moderate physical activities, respectively, compared with NAS [31]. A higher percentage of St-A who meet the physical activity guidelines is to be expected, considering that St-A typically have regular and compulsory training sessions and competitions.

Whilst moderate physical activity such as brisk walking or light cycling provides foundational benefits for general health and chronic disease prevention [32], vigorous physical activity such as running or intense aerobic exercise offers additional, unique advantages, particularly for cardiovascular health and longevity [33]. Given this close association, it is often assumed that these measures exist on a continuum, where individuals meeting vigorous physical activity guidelines are also expected to meet moderate physical activity thresholds. However, further analysis of the St-A category showed that among the 21 St-A men who did not meet the moderate physical activity guidelines, 13 still met the vigorous physical activity guidelines (Supporting Table 2). Similarly, of the 16 St-A women in this study who did not meet moderate physical activity guidelines, 10 still met the vigorous guidelines indicating that these categories are not overlapping but independent. These findings suggest that moderate and vigorous physical activities are not necessarily overlapping categories but represent distinct and independent patterns of behaviour in student populations. This distinction underscores the importance of considering moderate and vigorous physical activity as complementary components in health promotion strategies, rather than hierarchical or overlapping [33]. Promoting both types of activity in interventions ensures a comprehensive approach to physical activity that addresses the full spectrum of health benefits.

For St-A, these results suggest that achieving high levels of vigorous activity does not automatically translate into meeting moderate activity goals. This indicates a potential gap in activity diversity that could be targeted in tailored health and training programs. Tailored strategies for both St-A and NAS should focus on integrating moderate activities, such as recreational sports, alongside vigorous activities. Such a balanced approach accounts for behavioural preferences and ensures that individuals from both groups can achieve optimal health outcomes.

### 4.2. Nutrition

Athletes, because of their focus on optimal physical performance, are often perceived to have a good working knowledge of nutritional principles to maintain their health status. In addition to performance considerations, consuming a diet high in fruits and vegetables also plays an important role in reducing one's risk of developing NCDs [34]. However, despite growing awareness of the health benefits of fruit and vegetable consumption in the media and medical community [35], both St-A and NAS continue to exhibit low levels of fruit and vegetable intake. In accordance, our study shows that less than 10% of both groups consume five or more servings per day and that more than half of both groups consumed only one to two servings, showing that athletes are at an equally high risk of suboptimal nutritional practises compared to the NAS counterparts. This finding is not unique to our study as Cronin et al. showed that students in Gauteng consumed two fruit servings or less per week [21]. Furthermore, the South African research regarding the dietary habits of students from other provinces such as the Northern province [36], Eastern Cape [37] and Free State [38] had similar findings of poor fruit and vegetable consumption in student groups. Specific to the food environments of South African universities, students have claimed that fruits and vegetables are not easily available on campus [39, 40], a factor affecting St-A and NAS alike.

### 4.3. Smoking Habits

A higher percentage of daily or almost daily smokers was observed among NAS compared to St-A, whilst a larger proportion of St-A than NAS were nonsmokers. However, analyses per gender revealed that these findings were only evident among males; females displayed a similarly low prevalence of engagement in smoking behaviours. Our findings support other international literature which also reported a lower prevalence of smoking among St-A compared to NAS [41, 42]. Despite the paucity of recent data on St-A smoking habits, the former findings and those of the current study suggest that sports involvement at the university may factor against smoking. Specifically, St-A may be less willing to experiment with smoking behaviours due to the associated health risks [43] and commitment to success within their sport [42]. Importantly, university enrolment is a critical period for developing smoking habits [44, 45], with earlier initiation increasing the risk of long-term addiction and health consequences. Therefore, implementing smoking cessation interventions on university campuses is essential.

### 4.4. Alcohol Consumption

Despite the misconception that St-A would avoid alcohol consumption due to its deleterious effects on athletic performance [7, 8, 14], the current study observed a high prevalence of alcohol consumption among both St-A and NAS in which more than four-fifths of both groups consume alcohol. A comparable prevalence of alcohol consumption among St-A has been reported in Gauteng with 86% of male and 73% of female St-A consumed alcohol [21]. Another study by Surujlal et al. reported that male and female St-A consumed high and moderate quantities of alcohol [22]. The geographical location of this study with the strong historical wine culture found in the Cape Winelands may also have contributed to the similar alcohol consumption habits of St-A and NAS in this study. Moreover, sport holds a significant place in South African culture, and the close association between sport and alcohol [46] may further explain why the percentage of St-A consuming alcohol matched that of NAS; despite the potential effect, it may have on sports performances. Further investigation pertaining to the reasons for alcohol consumption is warranted among South African St-A and NAS.

### 4.5. Cluster Analysis

The cluster analysis in this study identified two clusters based on the investigated lifestyle risk behaviours: a higher risk cluster (inactive smokers) and a lower risk cluster (active nonsmokers) in which lifestyle-risk behaviours, including smoking, inadequate vigorous and moderate physical activity, and suboptimal nutritional practices co-occur. In addition, the findings of this study show that 35% of St-A are represented in the higher risk cluster, underscoring the necessity for enhanced screening and health information practices for all university students, including athletes. Importantly, in the current study, self-reported gender and age did not significantly contribute to the clustering solution. Age and gender as predictors of engaging in more than one lifestyle risk factors have previously been found to be inconsistent but older age and being female might be associated with fewer risk factors [18].

### 4.6. Perspective

Transitioning to tertiary education represents a significant milestone for students during which their newfound independence may predispose them to adopt behavioural risk patterns that increase their likelihood of developing lifestyle-related diseases, such as NCD's, later in life [47–49]. This study shows that South African university students engage in multiple lifestyle-related risk behaviours, regardless of their participation in a structured athletic programme. High rates of alcohol consumption (over 80%), inadequate fruit and vegetable intake (with fewer than 10% meeting daily recommendations) and a significant proportion of NAS failing to meet physical activity guidelines (approximately 50%–73%) remain a large concern. The co-occurrence of multiple lifestyle-related risk behaviours in this study highlights the need for a holistic health policy that considers the challenges of a low-resourced setting such as South Africa where health infrastructure and funding are limited. Specifically, cost-effective and scalable interventions that prioritise the development of targeted programmes addressing multiple risk behaviours concurrently are needed to effectively promote student health and well-being [50].

Among the St-A in this study, we observed inadequate fruit and vegetable intake, high rates of alcohol consumption (over 80%) and a notable proportion failing to meet physical activity guidelines for vigorous and moderate physical activity levels. Whilst these findings may not be generalisable to the entire tertiary education landscape, they carry significant clinical implications for coaches and team physicians responsible for the health and performance of St-A. St-A who present with one lifestyle-related risk behaviour should be screened for other co-occurring behaviours, as these can often cluster together. Tools completed annually, such as the MaRooN Health Passport, can play a pivotal role in identifying at-risk St-A and facilitate early intervention and targeted support from the teams' healthcare providers. In addition, greater emphasis should be placed on St-A education regarding nutrition and physical activity to minimise the risks of relative energy deficiency in sport (RED-S) and LEA [17], musculoskeletal conditions and cardiovascular diseases [51]. Incorporating regular assessments of these lifestyle-related risk behaviours into routine health evaluations will not only be important for optimising athletic performance and injury prevention but also for safeguarding the long-term health and well-being of St-A.

### 4.7. Strengths and Limitations

This study has several unique characteristics. Firstly, by employing a cluster analysis, this research provides a holistic view and nuanced understanding of how different behaviours cluster together in university-going adults, which is crucial for developing targeted interventions in this population group. Whilst separate cluster analysis models for St-A and NAS may highlight unique patterns and risk profiles for each group, combining St-A and NAS in a single cohort offers a comprehensive understanding of shared lifestyle-related risk behaviours across the student population. This approach facilitates direct comparison, aids in the development of inclusive interventions and enhances the statistical robustness of the analysis. Future studies employing larger sample sizes may be used to characterise separate models for St-A and NAS.

Moreover, we focussed on both St-A and NAS, where many studies tend to focus on either population individually. This approach allows for a comparative analysis that enriches the understanding of how athletic involvement influences lifestyle behaviours across different student groups. Lastly, focussing on a South African university setting adds a unique geographical and cultural context to the literature, which is often dominated by studies from high-income, Western countries. This aspect can enhance the diversity and inclusivity of global health research. Each of these elements contributes to the robustness and innovativeness of this study, positioning it as a valuable contribution to the fields of public health, behavioural science and educational policy.

The main limitations in this study design were the use of a self-reported questionnaire, which may introduce recall and social desirability bias, and also voluntary participation, which could result in self-selection bias. However, given that the observed behaviours align with trends from other tertiary institutions, we believe that these results reflect broader patterns in student lifestyle behaviours.

In addition, the authors acknowledge that a more detailed analysis on binge drinking and smoking behaviours would have strengthened the validity of the study. Even though there may be conflicting research about the health benefits of different types and amounts of alcohol use [51], no alcohol use has been found to have any performance benefit for athletes [52]. The number of cigarettes smoked per day was neither assessed among the current smokers nor did the questionnaire specifically reference the use of e-cigarettes.

The use of multivitamin supplementation was not assessed. However, it is important to note that vitamin and mineral supplements cannot replace the diverse and essential benefits provided by whole fruits and vegetables, including dietary fibre, phytonutrients and other bioactive compounds [53, 54]. The consistently low intake of fruits and vegetables observed in this study remains a critical concern and should be investigated in follow-up studies. Lastly, the St-A and NAS in this study were recruited from one university in the Western Cape, which restricts the generalisability of the results on a provincial and national level.

Although not a limitation per se, it should be noted that the data collection for this study coincided with the COVID-19 pandemic, and the associated restrictions implemented across South Africa during this time, may have influenced the participants' lifestyle behaviours, as outlined in the discussion.

## 5. Conclusion

The current study revealed that a similar percentage of South African St-A and NAS engage in certain lifestyle-related risk behaviours that may compromise their health, academics and sport performances. Specifically, more than 90% of both St-A and NAS consume insufficient servings of fruits and vegetables. In addition, a similarly high percentage of St-A (81.4%) and NAS (85.3%) consume alcohol regularly. The prevalence of physical inactivity was twice as high among NAS compared with St-A, although there is still a quarter of St-A who do not meet vigorous and moderate physical activity guidelines, likely due to the characteristics of certain sports. Whilst more male NAS than male St-A experimented with cigarettes, smoking habits were comparably low among females from both groups. The years spent at the university bring about a sense of autonomy where decisions about adopting behaviours are made, and many of these learned behaviours continue postgraduation into late adulthood. This suggests that university is an important period to encourage healthy behaviours and the avoidance of risk behaviours.

## Figures and Tables

**Table 1:** Prevalence and distribution of lifestyle-related risk behaviours in St-A and NAS.

	Males		*p* value^a^	Females		*p* value^b^	All		*p* value^c^	*p* value^d^
	NAS	St-A		NAS	St-A		NAS	St-A		
	*n* = 90	*n* = 91		*n* = 53	*n* = 54		*n* = 143	*n* = 145		
Alcohol use frequency										
Never	15.6% (14)	14.3% (13)	0.497	13.21% (7)	25.9% (14)	0.496	14.7% (21)	18.6% (27)	0.737	0.025
Monthly or less	20.0% (18)	15.4% (14)		32.08% (17)	31.5% (17)		24.5% (35)	21.4% (31)		
2–4 times per month	36.7% (33)	49.4% (45)		37.74% (20)	25.9% (14)		37.1% (53)	40.7% (59)		
2-3 times per week	23.3% (21)	16.5% (15)		15.09% (8)	14.8% (8)		20.3% (29)	15.9% (23)		
Daily or almost daily	4.4% (4)	4.4% (4)		1.89% (1)	1.9% (1)		3.5% (5)	3.4% (5)		
Smoking										
Never	62.2% (56)	80.2% (73)	0.022	77.4% (41)	83.3% (45)	0.882	67.8% (97)	81.4% (118)	0.020	0.523
Monthly or less	11.1% (10)	9.9% (9)		7.5% (4)	7.4% (4)		9.8% (14)	9.0% (13)		
2–4 times per month	4.4% (4)	4.4% (4)		3.8% (2)	3.7% (2)		4.2% (6)	4.1% (6)		
2-3 times per week	6.7% (6)	1.1% (1)		3.8% (2)	1.9% (1)		5.6% (8)	1.4% (2)		
Daily or almost daily	15.6% (14)	4.4% (4)		7.5% (4)	3.7% (2)		12.6% (18)	4.1% (6)		
Servings/day of fruit and vegetables^^^						0.488				
≥ 5	6.7% (6)	6.6% (6)	0.777	9.4% (5)	13.0% (7)		7.7% (11)	9.0% (13)	0.673	0.002
3-4 servings	23.6% (21)	29.7% (27)		45.3% (24)	42.6% (23)		31.7% (45)	34.5% (50)		
1-2 servings	61.8% (55)	58.2% (53)		41.5% (22)	44.4% (24)		54.2% (77)	53.1% (77)		
None	7.9% (7)	5.5% (5)		3.8% (2)	0.0% (0)		6.3% (9)	3.4% (5)		
Physical activity^#^										
Inadequate moderate	50.6% (43)	24.7% (21)	< 0.001	64.2% (34)	30.8% (16)	< 0.001	55.7% (78)	27.0% (37)	< 0.001	0.131
Inadequate vigorous	56.3% (49)	24.7% (21)	< 0.001	73.6% (39)	34.6% (18)	< 0.01	62.9% (88)	28.5% (39)	< 0.001	0.035

Abbreviations: NAS, nonathlete students; St-A, student athletes.

^a^ *p* value (*χ*^2^ test) for the comparison between NAS men and St-A men.

^b^ *p* value (from *χ*^2^ test test) for the comparison between NAS females and St-A females.

^c^ *p* value (from *χ*^2^ test test) for the overall comparison between all NAS and SA.

^d^ *p* value (from *χ*^2^ test test) for the gender comparison across both nonathlete and athlete groups.

^^^ *n* = 142 St-A who reported the number of servings of fruits and vegetables consumed per day (*n* = 89 male; *n* = 53 female).

^#^Vigorous and moderate physical activity data missing for *n* = 8 St-A (*n* = 6 male; *n* = 2 female) and *n* = 3 NAS males.

**Table 2:** Prevalence/distribution of health and lifestyle-related risk behaviours and demographic descriptors between Cluster 1 and Cluster 2.

	Cluster 1	Cluster 2	*p* value
	*n* = 156	*n* = 120	
	*n* (%)	*n* (%)	
Alcohol use frequency			
Never	22 (14.1%)	24 (20.0%)	0.073
Monthly or less	29 (18.6%)	35 (29.2%)	
2–4 times per month	68 (43.6%)	41 (34.2%)	
2-3 times per week	30 (19.2%)	18 (15.0%)	
Daily or almost daily	7 (4.5%)	2 (1.7%)	
Smoking status			
Never	85 (54.5%)	120 (100%)	0.001
Monthly or less	26 (16.7%)	0 (0%)	
2–4 times per month	12 (7.7%)	0 (0%)	
2-3 times per week	10 (6.4%)	0 (0%)	
Daily or almost daily	23 (14.7%)	0 (0%)	
Fruit/veg intake			0.009
≥ 5	10 (6.4%)	13 (10.8%)	
3-4 servings	43 (27.6%)	50 (41.7%)	
1-2 servings	93 (59.6%)	55 (45.8%)	
None	10 (6.4%)	2 (1.7%)	
Inadequate vigorous activity			0.001
Met goals	30 (19.2%)	120 (100.0%)	
Did not meet goals	126 (80.8%)	0 (0.0%)	
Inadequate moderate activity			0.001
Met goals	76 (48.7%)	86 (71.7%)	
Did not meet goals	80 (51.3%)	34 (28.3%)	
Demographic descriptors			
Gender			0.245
Male	92 (59.0%)	79 (65.8%)	
Female	64 (41.0%)	41 (34.2%)	
Athletic status			0.001
NAS	102 (65.4%)	37 (30.8%)	
St-A	54 (34.6%)	83 (69.2%)	
Age groups			
18–21	44 (28.2%)	33 (27.5%)	0.827
22–23	59 (37.8%)	51 (42.5%)	
24–26	39 (25.0%)	25 (20.8%)	
27+	14 (9.0%)	11 (9.2%)	

*Note:p* value (*χ*^2^ test) for the comparison between Cluster 1 and Cluster 2.

Abbreviations: NAS, nonathlete students; St-A, student athletes.

## Data Availability

The data that support the findings of the current study are available from the corresponding author upon reasonable request.

## Supporting Information

Supporting Table 1: Participants' sports participation in team sports, individual sports and other sport categories as indicated in the MaRooN Health Passport.

Supporting Table 2: Distribution of participants reporting adequate moderate and vigorous physical activity by gender and athlete status.
