# Supplementary material for: How Healthy Are Our Students Really? Lifestyle-Related Health Risk Behaviour Patterns in Student Athletes and Nonathlete Students
Source: Transl Sports Med. 2025 Mar 20;2025:1994649. doi: 10.1155/tsm2/1994649 (PMC11949602; doi:10.1155/tsm2/1994649)
Supplement: Supporting Information — Additional supporting information can be found online in the Supporting Information section. [file 1994649.f1.docx]

| **Supplementary Table 1. Participants’ sports participation in team sports, individual sports, and other sport categories as indicated in the MaRooN Health Passport.** | | | | | | |
| --- | --- | --- | --- | --- | --- | --- |
| **Category** |  | **St-A** | |  | **NAS** | |
|  |  |  | n= 145 |  |  | n=143 |
| **Team Sports** |  | **Participants (n)** | **Group total,**  **n (%)** |  | **Participants (n)** | **Group total,**  **n (%)** |
|  | Basketball | 11 |  |  | 2 |  |
|  | Cricket | 16 |  |  | 6 |  |
|  | Hockey | 22 |  |  | 6 |  |
|  | Netball | 7 |  |  | 3 |  |
|  | Rugby | 43 |  |  | 10 |  |
|  | Soccer | 16 |  |  | 14 |  |
|  | Water Polo | 7 |  |  | 1 |  |
|  |  |  | 122 (84%) |  |  | 42 (29%) |
| **Individual Sports** |  |  |  |  |  |  |
|  | Athletics | 12 |  |  | 8 |  |
|  | Cycling | 13 |  |  | 13 |  |
|  | Golf | 13 |  |  | 9 |  |
|  | Gymnastics | 1 |  |  | 1 |  |
|  | Swimming | 17 |  |  | 14 |  |
|  | Tennis | 3 |  |  | 13 |  |
|  | Judo | 1 |  |  | 1 |  |
|  | Surfing | 5 |  |  | 3 |  |
|  |  |  | 65 (45%) |  |  | 62 (43%) |
| **Other Activities** |  |  |  |  |  |  |
|  | CrossFit | 1 |  |  | 4 |  |
|  | Gym | 43 |  |  | 53 |  |
|  | Hiking | 7 |  |  | 28 |  |
|  | Running | 29 |  |  | 43 |  |
|  | Squash | 2 |  |  | 6 |  |
|  |  |  | 82 (56%) |  |  | 134 (94%) |
| St-A frequently reported being involved in more than one sporting type at university level. Participants were able to select more than sporting activity that they participated in. | | | |  |  |  |

| **Supplementary Table 2. Distribution of participants reporting adequate moderate and vigorous physical activity by gender and Athlete Status^#^** | | | | | | |
| --- | --- | --- | --- | --- | --- | --- |
|  | **NAS** | |  | **St-A** | |  |
|  | Adequate VPA | Inadequate VPA | Total: | Adequate VPA | Inadequate VPA | Total: |
| Males |  |  |  |  |  |  |
| Adequate MPA | 29.9% (26) | 19.5% (17) | 49.4% (43) | 60.0% (51) | 15.2% (13) | 75.3% (64) |
| Inadequate MPA | 13.8% (12) | 36.8% (32) | 50.6% (44) | 15.3% (13) | 9.4% (8) | 24.7% (21) |
| Females |  |  |  |  |  |  |
| Adequate MPA | 17.0% (9) | 19.0% (10) | 35.8% (19) | 46.2% (24) | 23.1% (12) | 69.2% (36) |
| Inadequate MPA | 9.4% (5) | 54.7% (29) | 64.2% (34) | 19.2% (10) | 11.5% (6) | 30.8% (16) |
| MPA, moderate physical activity. VPA, vigorous physical activity. St-A, student athletes; NAS, non-athlete students. #Vigorous and moderate physical activity data missing for n = 8 St-A (n = 6 male; n = 2 female) and n = 3 NAS males. Cut off points for inadequate moderate physical activity was <600 metabolic equivalent of task [MET] minutes per week and for inadequate vigorous physical activity <1500 MET minutes per week. | | | | | | |
